# Impact of the Genius Digital Diagnostics System on workflow and accuracy compared with the ThinPrep Imaging System for review of ThinPrep Papanicolaou tests

**DOI:** 10.1093/ajcp/aqaf099

**Published:** 2025-09-25

**Authors:** Kathleen M Murphy, Kristina Weatherhead, Carrie Chenault, Chinh Nguyen, Kari Sefcik, Sarah Harrington, Kasey Johnson, Yan Lemeshev

**Affiliations:** ProPath, A Sonic Healthcare Anatomic Pathology Practice, Dallas, TX, United States; ProPath, A Sonic Healthcare Anatomic Pathology Practice, Dallas, TX, United States; ProPath, A Sonic Healthcare Anatomic Pathology Practice, Dallas, TX, United States; ProPath, A Sonic Healthcare Anatomic Pathology Practice, Dallas, TX, United States; ProPath, A Sonic Healthcare Anatomic Pathology Practice, Dallas, TX, United States; Scientific Affairs Department, Hologic, Inc, Marlborough, MA, United States; Scientific Affairs Department, Hologic, Inc, Marlborough, MA, United States; ProPath, A Sonic Healthcare Anatomic Pathology Practice, Dallas, TX, United States

**Keywords:** digital cytology, digital pathology, artificial intelligence, cervical cancer, cytology, cytopathology, workflow, lab efficiency, Papanicolaou test, Genius Digital Diagnostics System

## Abstract

**Objective:**

In this study, we compared the workflow of the Genius Digital Diagnostics System (Hologic, Inc) with our current workflow based on the ThinPrep Imaging System (Hologic, Inc) to assess potential efficiencies associated with digitalization of Papanicolaou screening.

**Methods:**

Each step of the current workflow (glass slide movement and slide review) and the experimental workflow were documented. Substantial workflow efficiencies were associated with the reduction of glass slide movement observed with the experimental workflow of the Genius system compared with the ThinPrep system.

**Results:**

The ThinPrep-based workflow required more than 5 hours of hands-on time at specific synchronized times throughout the day, whereas the hands-on time of the experimental Genius Digital Diagnostics System was just over an hour and allowed glass movement at flexible times. In addition to these workflow efficiencies, the Genius Digital Diagnostics System resulted in much shorter review times (70.1 seconds) than the ThinPrep Imaging system (138.0 seconds) while maintaining similar agreement to the sign-out diagnosis.

**Conclusions:**

This study demonstrated that implementing a Genius Dx-based workflow may result in substantial efficiency gains, which can mitigate workforce shortages and improve turnaround time without compromising screening accuracy.

**Key Points**

- Papanicolaou testing is integral to cervical screening, but dependence on glass slides and labor shortages are critical challenges. Digital cytology technology may address these issues.
- Innovation in the field of cytology—specifically, digital cytology with an AI algorithm—can reduce current workflow inefficiencies in cervical cancer screening.
- This study demonstrated that the Genius Digital Diagnostics System substantially increased workflow efficiencies while maintaining strong concordance for disease detection.

## INTRODUCTION

The Papanicolaou test has been an integral part of cervical cancer screening since its introduction in the 1940s, substantially reducing cervical cancer morbidity.^1^ Over the past 30 years, there have been notable improvements in the Papanicolaou test. In the 1990s, liquid-based cytology (LBC) was introduced, with ThinPrep (Hologic, Inc) and BD SurePath (Becton Dickinson) systems. Several advantages of LBC have been documented in the literature, including improvements in reproducibility, sample quality, disease detection, screening efficiency, and the ability to perform out-of-the-vial molecular testing.^2^

The field of cytology has continued to innovate to meet the needs of the field and optimize workflow. Hologic’s ThinPrep Imaging System (TIS) was approved in 2003, and Becton Dickinson’s BD FocalPoint GS imaging system was approved in 2008, incorporating image analysis algorithms to detect areas of interest on a Papanicolaou slide for further review, resulting in increased screening efficiency while maintaining diagnostic accuracy.^3,4^ Multiple studies have evaluated the time efficiencies of slide review using the TIS compared with the predicate screening mechanisms, such as conventional cytology or manual screening of LBC, where all demonstrated workflow improvements of using the TIS resulted in increased screening efficiencies while maintaining diagnostic accuracy.^5-7^

Advances in digital imaging and artificial intelligence (AI) have allowed for further innovation to aid cervical cancer screening. Even with the use of the ThinPrep Imaging or FocalPoint systems, cytology methodology can be labor intensive for cytologists and pathologists. In addition, there has been a decline in new cytologists entering the workforce. Recent data show a 38% decrease in cytologists taking the American Society for Clinical Pathology GYN Proficiency Test from 2005 to 2022,^7^ which has affected laboratory personnel and turnaround time. The Papanicolaou test currently relies on the movement of a physical glass slide, which has some potential challenges, such as time and logistics of slide retrieval and transportation, lost or broken slides, and cost and physical storage of archived slides.

A potential solution is the use of digital cytology and whole-slide imaging with AI. Automation in the cytology field has been introduced to increase the efficiency and objectivity of interpretation of cervical disease during screening. This increase can be achieved by coupling automation of slide screening with an AI algorithm to detect abnormal cells. Whole-slide image scanners digitize whole glass slides, creating large data files that can then be interrogated by AI screening algorithms. Whole-slide imaging has been used previously in digital pathology^8,9^; however, adoption of digital cytology has been slower due to technical challenges in imaging these samples. Digital scanners were developed predominately for histology and use a single plane of focus. Due to the 3-dimensional nature of cells, digital imaging of cytology requires multiple focal planes to generate an in-focus image. To address these concerns, a new US Food and Drug Administration (FDA)–cleared digital cytology system that consists of a digital scanner with volumetric imaging technology and an AI algorithm was developed to aid in detection of cervical abnormalities and increase efficiency. In this study, we compared the workflow and screening accuracy of the Hologic Genius Digital Diagnostics System (Genius Dx) with the current standard of care, the TIS, in a high-volume laboratory.

## METHODS

This noninterventional study was conducted in 2 parts: a direct observation of glass slide logistics and a measurement of screening efficiency and accuracy for the Genius Dx and TIS systems compared with the standard-of-care clinical sign-out cytology result. Both study phases occurred on-site at a large commercial reference laboratory in the United States.

### Instrumentation

The ThinPrep Imaging System is an FDA-approved device that uses computer imaging technology to assist in primary cervical cancer screening.^10^ The ThinPrep system selects 22 fields of view (FOVs) for a cytologist to review. The system was used following the manufacturer’s instructions. Following the review of the 22 FOVs, the cytologist will either complete the diagnosis if everything is normal or review the entire slide if any abnormalities are identified in the 22 FOVs.

The Genius Digital Diagnostics System is a digital cytology system FDA cleared to aid in cervical cancer screening. It includes the Digital Imager, the Cervical AI algorithm, the Image Management Server, and the Review Station.^11^ The Genius Digital Imager creates high-resolution 2-dimensional images of ThinPrep slides composed of the most in-focus pixels from multiple focal planes using volumetric imaging technology. The Genius Cervical AI analyzes all the cells in the image and displays the clinically relevant cells in a tiled gallery that can be viewed on the Genius Review Station.

### Slide logistics workflow

The current-state TIS workflow study of slide logistics was completed by Nexus Global Solutions, Inc., an independent workflow consulting firm for diagnostic laboratories (Nexus, Plano, TX). The Nexus team observed and documented the physical movement, handling, storage, and management of clinical Papanicolaou cytology slides (test slides to quantify the hands-on time for the slide logistics processes. The slide logistics included 4 main steps: (1) slide Imaging, including loading and unloading slides; (2) transport to cytologists, including case load management, slide distribution, and postscreening workflow; (3) transport to pathologists, including case load management, slide distribution, and postscreening workflow; and (4) slide archiving and retrieval. At each of these steps, the hands-on time was calculated and compared with the experimental workflow for the digital cytology system.

### Screening workflow

The Genius Dx was evaluated by comparing it with the current TIS-based workflow. A sample size calculation was performed using the Statulator calculator to establish the minimum number of cases necessary to achieve the desired statistical power.^12^ The calculation was based on the abnormal diagnosis rate because this is the most clinically relevant confirmation of diagnostic accuracy for testing the digital cytology system. We estimated that an abnormal detection rate up to 2% higher than the current method would be acceptable, with a greater than 2% shift in abnormal cytology detection as a clinically meaningful difference. Because the laboratory reported an average of 10% abnormal cytology cases, a Genius Dx–based abnormal rate greater than 12% would be deemed clinically significant. Assuming a 95% CI (5% type I error) and 90% power (10% type II error), using a 1-tailed test. Because we would be testing whether the Genius Dx had a higher abnormal rate than the current methods, with a correlation of .7 between the pairs (estimated based on the 10% and 12% abnormal detection rates), the study would require a sample size of 1321 pairs to achieve a power of 90% and a 1-sided statistical significance of 5% for detecting a difference of 0.02 between marginal proportions. A total of *n* = 1500 sequential cases collected from the normal workflow over a 1-week period were included in the study and were reviewed by 1 of 2 sets of cytotechnologist-pathologist pairs using TIS and Genius Dx. The study had 2 study arms, where 750 cases were first reviewed by a cytotechnologist-pathologist pair using TIS followed by the Genius Dx after a 2-week washout, while the other cytotechnologist-pathologist pair first reviewed the cases using a Genius system followed by the ThinPrep system after a 2-week washout period. Cases assessed as negative for intraepithelial lesion or malignancy (NILM) were signed out and not sent to the pathologist for review, while NILM-reactive, atypical squamous cells of undetermined significance (AS-CUS), or more severe disease were sent to the pathologist for final sign-out, as is done in clinical practice. For the TIS review, the first cytologist-pathologist pair included 77 pathologist reviews, and the second cytologist-pathologist pair included 76 pathologist reviews. For the Genius Dx review, the first cytologist-pathologist pair included 131 pathologist reviews, and the second cytologist-pathologist pair included 116 pathologist reviews.

### Data analysis

**Figure 1:**
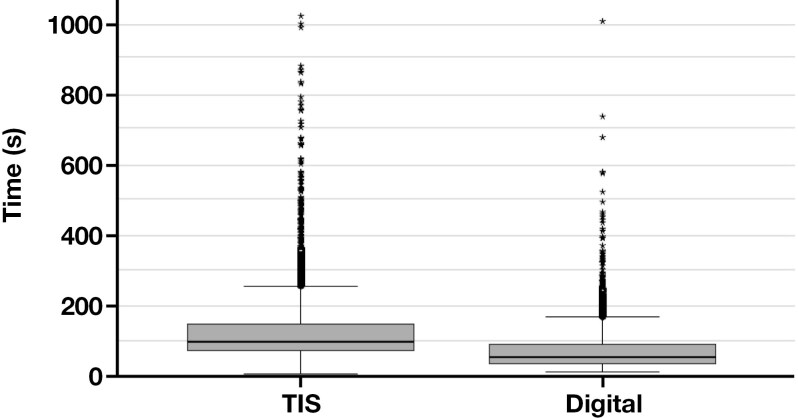
Box plot for the overall review time for each case, including cytologist and pathologist review (pathologist review time was added to the cytologist assessment time when applicable). Not pictured are 15 ThinPrep Imaging System (TIS) cases with review time >1000 seconds and 3 digital cases with review time >1000 seconds.

Review efficiency was measured as the amount of time for Genius Dx or TIS review. Because time was calculated automatically based on when a diagnostic assessment was entered for each case, the data were explored for outliers to exclude artificially long times that would indicate that a reviewer had stepped away from their workstation or had a prolonged non–study-related distraction. A *z* score was calculated (review time minus mean review time divided by the SD of review times), and scores greater than 3 were flagged as outliers and excluded from efficiency calculations. The outliers for cytologist reviews within the TIS arm included an elapsed time of 783 seconds or more (21 cases excluded) and within the Genius Dx arm included 429 seconds or more (12 cases excluded). The outliers for pathologists included 1 case in the TIS arm that was 81 807 seconds and 5 cases in the Genius Dx arm that exceeded 112 seconds. Figure 1 shows a box plot of overall case time for each process; cytologist assessment time was added to a pathologist review assessment if a review was provided. A *z* score was calculated (review time minus mean review time divided by the SD of review times), and scores greater than 3 were flagged as outliers and excluded from efficiency calculations.

For a total case time calculation, if either the cytologist or pathologist time exceeded a z score of 3, the case was excluded from the time comparison within that study arm (resulting in 22 cases excluded from the TIS arm and 17 cases excluded from the Genius Dx). The average number of seconds spent on diagnostic assessment using the Genius Dx vs the TIS were compared using an independent *t* test.

Statistical significance was assumed at *P* < .05. Analyses were performed using IBM SPSS Statistics 27. Each review arm (Genius Dx and TIS) was compared with the original clinical sign-out diagnosis, which was treated as the true diagnosis for the case. The NILM agreement, abnormal agreement (diagnostic assessments of AS-CUS or more severe disease), and overall agreement (unsatisfactory, NILM, or AS-CUS or worse) were calculated to determine whether the Genius Dx is as diagnostically accurate as the ThinPrep system, particularly with the detection of abnormal diagnoses. The Pearson χ^2^ test was used to determine whether the proportion of diagnostic categories was different based on diagnostic assessment modality (Genius Dx or TIS).

## RESULTS

### Slide logistics workflow

**Figure 2:**
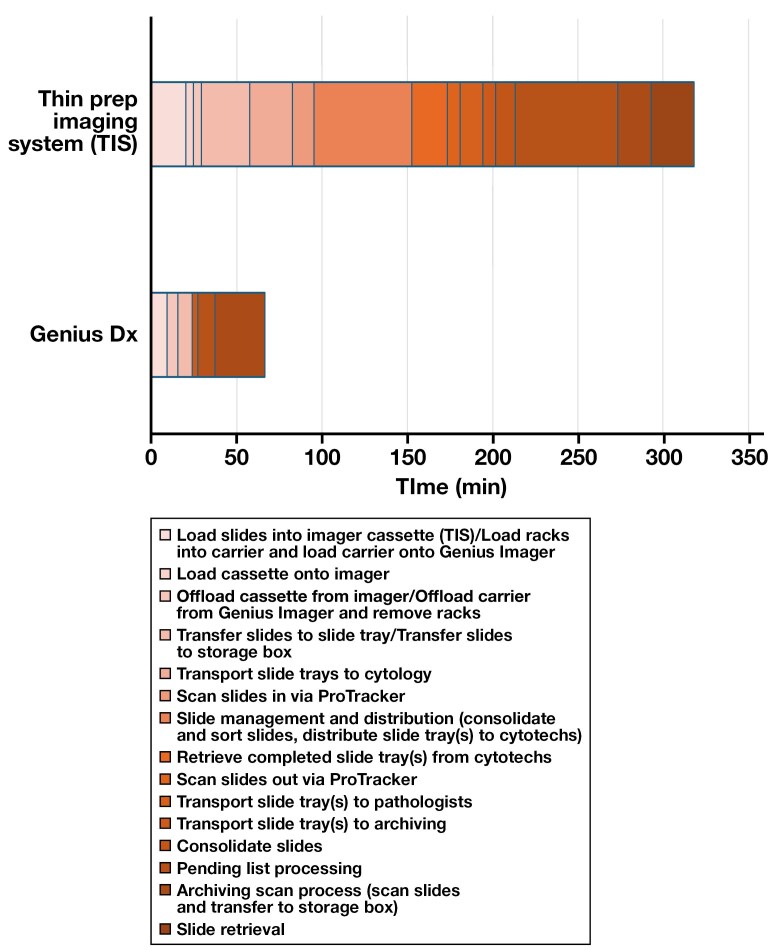
Graphical depiction of the overall hands-on time for each simulated workflow (ThinPrep Imaging System [TIS] and Genius Digital Diagnostics System [Genius Dx]), broken out by each daily task.

The hands-on times of the current workflow (TIS) and experimental (Genius Dx) workflow were observed and measured for the movement of glass slides for the different daily tasks, and then further estimated for a full workday. The hands-on time for the 4 main steps were as follows: (1) slide imaging, (2) transport to cytologist, (3) transport to pathologists, and (4) slide archiving and retrieval. Slide preparation and staining were not included in the analysis because the process is identical for both workflows. Because of the differences in workflows and batches between the systems (25 slides per cassette for TIS vs 40 slides per carrier for the Genius Dx), the hands-on time analysis was upscaled to daily hands-on time for comparisons. Each task of moving glass slides in the current and experimental workflows was observed and measured; the hands-on time for these movements is displayed in Figure 2. In addition to the total hands-on time, the TIS workflow has additional caveats that require more synchronization and time.

Because Genius Dx images are available digitally, there were no hands-on time measurements for transport to cytologists or transport to pathologists. The hands-on time was pertinent only for the slide imaging and slide archiving and retrieval steps in the experimental Genius Dx workflow. The overall hands-on time of the glass slide with the current TIS workflow is approximately 5 hours, 18 minutes daily compared with the Genius Dx workflow hands-on time of 1 hour, 6 minutes daily (Figure 2). This experimental Genius Dx workflow resulted in a 79% reduction in hands-on time of the glass slide compared with the current TIS workflow. In addition to the reduction in hands-on time with the Genius Dx compared with the TIS, there is more flexibility in the overall tasks with the Genius Dx than with the TIS, which requires specific synchronization of the workflow to move glass slides to cytologists and pathologists for review in a timely fashion.

### Screening efficiency and accuracy

**Figure 3:**
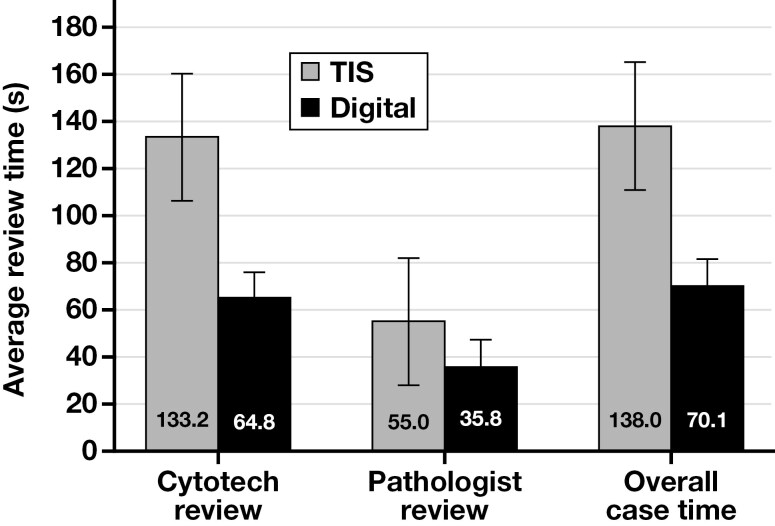
Average review times of cytologists, pathologists, and overall case review time on the ThinPrep Imaging System (TIS) compared with the Genius Digital Diagnostics System (Genius Dx).

Review times for the TIS and Genius Dx systems were measured using a simulated laboratory information system that captured the amount of time from when the case was opened until a diagnosis was submitted (summarized in Figure 3). Cytologists spent much more time reviewing slides on the TIS (mean [SD], 133.2 [109.8] seconds) than on the Genius Dx (mean [SD], 64.8 [54.6] seconds; *t*_2164.4_ = 21.5; *P* < .001). Pathologists also spent much more time reviewing slides using TIS (mean [SD], 55.0 [72.6] seconds) than on the Genius Dx (mean [SD], 35.8 [19.1] seconds; *t*_164.2_ = 3.2; *P* = .002). The total average time per case was much higher using TIS (mean [SD], 138.0 [123.1] seconds) than using the Genius Dx (mean [SD], 70.1 [59.7] seconds; *t*_2145.6_ = 19.0; *P* < .001) (Figure 3).

Table 1 shows the diagnostic distribution of the 1500 cases included in the screening efficiency and accuracy portion of the study. As typically observed in a screening population, the majority (93.5%) of cases were negative, which included negative, negative for *Actinomyces*-like organisms, negative for *Candida*, negative for *Trichomonas vaginalis*, and negative for endometrial cells in patients aged 45 years or older. A total of 87 cases in the study had a final clinical cytology diagnosis of AS-CUS; low-grade squamous intraepithelial lesion; atypical squamous cell, cannot exclude high-grade squamous intraepithelial lesion (ASC-H); high-grade squamous intraepithelial lesion (HSIL); and atypical glandular cell (AS-CUS or worse). Eleven cases were identified as unsatisfactory.

**Table 1:** Breakdown of the Clinical Sign-Out Diagnosis of the Study Cases

Diagnostic category	No.	% (*n* = 1500)
Unsatisfactory	11	0.70
Negative	1402	93.50
AS-CUS	56	3.70
Low-grade squamous intraepithelial lesion	23	1.50
ASC-H	2	0.10
HSIL	4	0.30
Atypical glandular cell	2	0.10

Abbreviations: ASC-H, atypical squamous cell, cannot exclude high-grade squamous intraepithelial lesion; AS-CUS, atypical squamous cells of undetermined significance; HSIL, high-grade squamous intraepithelial lesion.

For cases reviewed on TIS, the overall agreement was 94.8% (1422/1500 cases), and the exact diagnostic match was 93.3% (1399/1500 cases) compared with the original clinical sign-out diagnosis. Overall, 6.3% of the cases had a diagnosis of AS-CUS or worse using the TIS compared with the original clinical sign-out. For cases reviewed on the Genius Dx, the overall agreement was 91.5% (1372/1500 cases), and the exact diagnostic match was 89.9% (1348/1500 cases). Overall, 10.0% of the cases had a diagnosis of AS-CUS or worse using the Genius Dx compared with the original clinical sign-out. Despite more cases being called abnormal (and requiring pathologist review) using the Genius Dx vs TIS, the total time (cytologist and pathologist) to review all cases with the Genius Dx was 28.5 hours compared with just over 56 hours with TIS.

The overall agreement between case diagnosis and sign-out diagnosis was statistically significantly higher for TIS than for the Genius Dx ($\chi \frac{2}{1}$ = 13.0; *P* < .001); however, there was no statistically significant difference in agreement for AS-CUS or worse cases between the Genius Dx and TIS compared with the original sign-out diagnosis ($\chi \frac{2}{1}$ = 2.3; *P* = .126). The agreement for negative cases was statistically significantly higher for the TIS (96.1%) than for the Genius Dx (92.0%) compared with the sign-out diagnosis ($\chi \frac{2}{1}$ = 21.5; *P* < .001) (Table 2).

**Table 2:** Diagnostic Agreement of TIS and Genius Dx Reviews With the Original Sign-Out Diagnosis

	TIS			Genius Dx			χ^2^	*df*	*P* value
	*n*	Total No.	%	*n*	Total No.	%			
Overall agreement	1422	1500	94.8	1372	1500	91.5	13	1	<.001
NILM agreement	1348	1402	96.1	1290	1402	92.0	21.5	1	<.001
Abnormal agreement (AS-CUS or worse)	66	87	75.9	74	87	85.1	2.3	1	.126

Abbreviations: AS-CUS, atypical squamous cells of undetermined significance; NILM, negative for intraepithelial lesion or malignancy.

## DISCUSSION

Since its introduction, the Papanicolaou test has drastically decreased the incidence of cervical cancer worldwide.^1^ Currently in the United States, the gold standard for cervical cytology is image-guided Papanicolaou testing. A new approach to cytology uses digital imaging and an AI algorithm, which can modernize and improve workflow efficiencies of the Papanicolaou test; however, although digital imaging and AI algorithms have been used in histopathology,^8,9^ their use in cytology has been limited.

Hologic’s Genius Dx received FDA clearance in 2024 and is intended to aid in cervical cancer screening for the presence of atypical cells; cervical neoplasia, including its precursor lesions; and carcinoma from a scanned digital image presented in gallery format.^11^

Although our study is an early evaluation of these potential workflow benefits, other published studies have focused on the diagnostic performance of the Genius Dx.^13-15^ A recent validation study found that compared with a manual cytology review, the Genius Dx had higher agreement with the sign-out cytology diagnosis and more efficient screening.^13^ In addition, a large, retrospective study published in 2023 demonstrated that using the Genius Dx resulted in higher sensitivity for ASC-H and HSIL or worse disease and shorter review times than the TIS.^13^

While other studies^^13-15^^ have demonstrated the diagnostic performance of the Genius Digital Diagnostics System, our study is the first to specifically evaluate the potential workflow efficiencies for a laboratory transitioning to digital cytology. We demonstrated that the Genius Dx showed statistically significant improvement in laboratory efficiencies and required substantially less hands-on time than TIS in a head-to-head simulated workflow. The Genius Dx required less movement of glass slides from different areas within the laboratory, with a 79% reduction in hands-on time, and provided additional flexibility in the cytology workflow. In addition, because the Genius Dx is not tied to the movement of the glass slide, it does not require the same time synchronization of the workflow. These improvements and flexibility in workflow could aid in the current climate of laboratory personnel shortages.

In addition, the Genius Dx allows for substantial efficiency improvements associated with reviewing and signing-out cases. This finding is reflected in cytologists and pathologists reviewing cases in less time on the Genius Dx than on TIS. There was no statistically significant difference in disease detection (AS-CUS or worse) between the 2 systems and the original clinical sign-out diagnosis. The NILM agreement with the original diagnosis was higher for the TIS than for the Genius Dx. As a result, the overall agreement with the original sign-out diagnosis was higher for the TIShin than for the Genius Dx. Although the exact reason for the lower NILM agreement with the Genius Dx is unknown, it may be due in part to a lack of experience and the learning curve associated with using a new technology, resulting in lower specificity when adopting new technologies. Previous research has demonstrated this phenomenon and noted that this decrement in specificity is overcome when users become familiar with the technology.^16^

Previous studies published when TIS was implemented demonstrated that laboratories gained substantial improvements in efficiency over manual screening^16,17^. One study showed a 42% reduction in review time using TIS compared with manual review.^16^ With TIS, the glass slide is imaged and the reviewer is directed to 22 FOVs that contain cells or cell clusters of interest. If all the cells in these FOVs are normal, the slide can be signed out as normal, and the reviewer can move to the next case. If there are any suspicious or abnormal cells, the reviewer is required to rescreen the full slide manually. In the case of the Genius Dx, the reviewer can interpret the diagnosis from the gallery, providing review-time efficiencies. The gallery approach may be particularly useful for sign-out of abnormal cases because pathologists can see a representation of the entire case and any annotations without having to move around the glass slide to locate the cytologist’s marked cells.

The current study was limited to an experimental scenario before implementation of the Genius Dx for clinical use and did not include every cervical cytology activity required within the laboratory. For example, reprocessed slides and slides that could not be imaged on either the TIS or Genius Dx were removed from the study set, cases did not go through a simulated quality control process, and cytologists and pathologists did not use their current laboratory information system and did not have access to standard clinical information. When the Genius Dx has been implemented in clinical practice, we expect differences in efficiencies compared with what we observed in this study. The additional tasks that we could not study will be documented in future papers when the Genius Dx has been implemented for routine screening. The cytologists and pathologists in this study were not using the Genius Dx in their current workflow and were not as familiar with it as they are with the TIS workflow, which may have resulted in capturing some of the learning curve rather than the full efficiencies and accuracy that could be expected once users are completely comfortable with the system. The cases included in this study were true screening cases in chronological order, with a limited number of abnormal cases, and histology correlation was not available at the time of analysis because these cases were selected from the current workflow. Additional studies are needed to expand on these findings.

This study is the first US study to demonstrate the benefits of the Genius Digital Diagnostics System for clinical workflow efficiencies. In a simulated workflow of the Genius Dx compared with the current standard of care (TIS), Genius Dx demonstrated a statistically significant improvement in the efficiency of the slide workflow and the screening (review, analysis) workflow while maintaining similar disease detection rates.
